# Editing of phosphatidic acid and phosphatidylethanolamine by acyl-CoA: lysophospholipid acyltransferases in developing *Camelina sativa* seeds

**DOI:** 10.1007/s00425-020-03408-z

**Published:** 2020-06-10

**Authors:** Sylwia Klińska, Katarzyna Jasieniecka-Gazarkiewicz, Kamil Demski, Antoni Banaś

**Affiliations:** Intercollegiate Faculty of Biotechnology, University of Gdansk and Medical University of Gdansk, Abrahama 58, 80–307 Gdansk, Poland

**Keywords:** False flax, LPAAT, LPEAT, Phosphatidylcholine, Phospholipid remodelling, Seed lipids

## Abstract

**Main conclusions:**

The main source of polyunsaturated acyl-CoA in cytoplasmic acyl-CoA pool of Camelina sativa seeds are fatty acids derived from phosphatidylcholine followed by phosphatidic acid. Contribution of phosphatidylethanolamine is negligible.

**Abstract:**

While phosphatidylethanolamine (PE) is the second most abundant phospholipid, phosphatidic acid (PA) only constitutes a small fraction of *C. sativa* seeds’ polar lipids. In spite of this, the relative contribution of PA in providing fatty acids for the synthesis of acyl-CoA, supplying cytosolic acyl-CoA pool seems to be much higher than the contribution of PE. Our data indicate that up to 5% of fatty acids present in mature *C. sativa* seeds are first esterified with PA, in comparison to 2% first esterified with PE, before being transferred into acyl-CoA pool via backward reactions of either acyl-CoA:lysophosphatidic acid acyltransferases (*Cs*LPAATs) or acyl-CoA:lysophoshatidylethanolamine acyltransferases (*Cs*LPEATs). Those acyl-CoAs are later reused for lipid biosynthesis or remodelling. In the forward reactions both aforementioned acyltransferases display the highest activity at 30 °C. The spectrum of optimal pH differs for both enzymes with *Cs*LPAATs most active between pH 7.5–9.0 and *Cs*LPEATs between pH 9.0 to 10.0. Whereas addition of magnesium ions stimulates *Cs*LPAATs, calcium and potassium ions inhibit them in concentrations of 0.05–2.0 mM. All three types of ions inhibit *Cs*LPEATs activity. Both tested acyltransferases present the highest preferences towards 16:0-CoA and unsaturated 18-carbon acyl-CoAs in forward reactions. However, *Cs*LPAATs preferentially utilise 18:1-CoA and *Cs*LPEATs preferentially utilise 18:2-CoA while catalysing fatty acid remodelling of PA and PE, respectively.

## Introduction

Fatty acid composition of membrane phospholipids is one of the key determinants of the physical properties of cell membranes. All acyl chains for membrane and storage lipid synthesis in plants are de novo produced in plastids by acyl carrier protein-dependent fatty acid synthesis (Ohlroge et al. 1979; Ohlrogge and Browse 1995). The ACP is then hydrolysed and the free fatty acids are transported to the cytosol across the plastid envelope and activated to acyl-CoA (Pollard and Ohlrogge 1999). These acyl groups are then utilised by different acyltransferases to synthesise principal phospholipids and triacylglycerol (TAG) in the process described by Kennedy (1961). The Kennedy pathway begins with acylation of glycerol-3-phosphate (G3P) by two acyltransferases: acyl-CoA:glycerol-3-phosphate acyltransferase (GPAT) and acyl-CoA:lysophosphatidic acid acyltransferase (LPAAT) to create phosphatidic acid (PA). PA is subsequently dephosphorylated by phosphatidic acid phosphatase to generate diacylglycerol (DAG), which is a substrate for storage and membrane lipid biosynthesis. DAG may be then acylated at the *sn*-3 position by diacylglycerol acyltransferase (DGAT) to produce triacylglycerol. DAG can be also converted into phosphatidylcholine (PC) by CDP-choline:1,2-diacylglycerol cholinephosphotransferase (CPT), which utilises DAG and CDP-choline as substrates. Phosphatidylethanolamine (PE) synthesis proceeds in a similar way to the PC synthesis the active form of ethanolamine (CDP-ethanolamine) interacts with DAG in the reaction catalysed by CDP-ethanolamine:1,2-diacylglycerol ethanolaminephosphotransferase (Kennedy and Weiss 1956).

Continuous exchange of acyl residues, especially in the *sn-2* position of the glycerol core of phospholipids (called lipid remodelling or acyl editing) takes place after the de novo phospholipid synthesis. The phospholipid acyl editing cycle involves rapid deacylation of phospholipids, which generates lysophospholipids and releases fatty acids (FA) or acyl-CoAs to the cytosol, followed by reacylation of the formed lysophospholipids.

Originally, the deacylation step was considered to be the function of phospholipases, especially taking into account the activity of phospholipase A2. The reacylation step of created lysophospholipids is done by acyl-CoA:lysophospholipids acyltransferases (LPLATs). These enzymes use different acyl-CoA to create phospholipid molecules with new acyl chains (but with the same phospholipid backbone). This cyclical process was described for the first time by Lands (1958) and is known as Lands’ cycle. Remodelling of, e.g., phosphatidylcholine during the process of synthesis of some unusual fatty acids like ricinoleic acid (12-hydroxy-cis-9-octadecenoic acid) or vernolic acid (cis-12,13-epoxy-cis-9-octadecenoic acid) can be conducted this way (Bafor et al. 1991, 1993; Banaś et al. 1997).

Besides the Lands’ cycle an alternative acyl-editing route exists in plants. In this cycle (we call it “LPLAT cycle”) the reversibility of the reaction catalysed by LPLAT enzymes plays the central role both deacylation of phospholipids and reacylation of formed lysophospholipids are performed by these enzymes. The involvement of LPLAT in exchange of fatty acids between PC and the pool of cytoplasmic acyl-CoA was suggested many years ago based on in vitro studies on microsomes isolated from soybean and safflower seeds (Stymne and Glad 1981; Stymne and Stobart 1984). Since then, the exchange of fatty acids between phospholipids and the pool of cytoplasmic acyl-CoA has been demonstrated many times in vivo and the involvement of LPLATs in this process has been postulated (Griffiths et al. 1988; Williams et al. 2000; Bates et al. 2007, 2013; Pan 2015). After cloning the genes encoding acyl-CoA:lysophosphatidylcholine acyltransferases (LPCATs), a member of LPLAT family, from different plant species, Lager et al. (2013) proved that these enzymes can operate both in forward and backward reactions; in forward reaction they synthesise PC from lysophosphatidylcholine (LPC) and acyl-CoA and in the backward reaction they transfer the acyl group from PC (especially *sn*-2 position) to acyl-CoA pool and create LPC. Reacylation of the formed LPC with a new acyl-CoA may then occur (forward reaction). Later on, Jasieniecka-Gazarkiewicz et al. (2016) demonstrated that remodelling of other phospholipids (PE, PA) can also take place in such a cycle and that this process can be catalysed by acyl-CoA:lyshosphatidylethanolamine (LPEAT) and acyl-CoA:lysophosphatidic acid acyltransferases—other members of LPLAT family.

The relative contribution of Lands’ cycle and LPLAT cycle in in vivo lipids remodelling has not been elucidated so far. Recently Klińska et al. (2019) provided some evidence concerning remodelling of PC of *Camelina sativa* seeds’ microsomal fractions via LPCAT (LPLAT cycle) and other enzymes’ action (associated with Lands’ cycle) and showed that the intensity of both cycles varies during seed development with LPLAT cycle being the dominating one. In the presented research we aim to: (1) characterise the biochemical properties of LPAAT and LPEAT-type of enzymes in developing *C. sativa* seeds; (2) characterise the substrate specificity of these enzymes in forward and backward reactions; (3) evaluate the possible contributions of PE and PA in providing fatty acids for acyl-CoA synthesis (which supplies cytosolic acyl-CoA pool available for different lipid biosynthesis, inter alia triacylglycerols) via backward reaction of LPLAT type of enzymes and via other reaction types (Lands cycle) and estimate the time of complete fatty acids turnover in PC, PE and PA of *C. sativa* seeds.

## Materials and methods

### Chemicals

The [1-^14^C]-labelled fatty acids were obtained from Perkin-Elmer Life Science (Waltham, MA, USA). The non-radioactive fatty acids, *sn*-1–18:1-lysophosphatidylethanolamine and *sn*-1–18:1-lysophosphatidic acid were purchased from Larodan Fine Chemicals (Malmö, Sweden). The [1-^14^C]-labelled acyl-CoAs and non-radioactive acyl-CoAs were synthesised according to the modified method described by Sánchez et al. (1973). The standards for thin-layer chromatography were purchased from Avanti Polar Lipids (Alabaster, AL, USA). All the other chemicals were obtained from Sigma Aldrich (St. Louis, MO, USA) or Merck (Darmstadt, Germany).

### Plant materials

Seeds of *Camelina sativa cv.* Suneson were planted in soil and cultivated in growth chamber at a constant temperature of 23 °C with 60% relative humidity with a photoperiod of 16 h of light (120 µmol photons m^−2^ s^−1^)/8 h of darkness. After 4–5 weeks, when plants had well-developed flower buds, each plant was marked and depending on the conducted experiment, the seeds were collected after the predefined time elapsed. For lipid analysis the seeds were harvested at 10, 17, 24, 31 and 60 DAF, whereas for microsomal preparation the seeds were collected at 17, 24 and 31 DAF. The dry weight of the seeds was approximately 0.14, 0.5, 0.7, 1.1 and 1.4 mg/seed at 10, 17, 24, 31 and 60 DAF, respectively (more detail in Klińska et al. 2019).

### Microsomal preparation and enzymes assays

Microsomal fractions were prepared from freshly harvested seeds at 17, 24 and 31 DAF, when it was possible to manually separate the embryo from the seed coat. The microsomes were prepared according to the method previously described by Stymne and Stobart (1984). Collected seeds were homogenised briefly with 0.1 M potassium phosphate buffer containing 1 mg/ml BSA, 0.33 M saccharose and catalase (1000 U/ml). The homogenates were filtered through Miracloth, diluted with homogenising buffer to 20 ml and centrifuged at 20,000×*g* for 12 min. The obtained supernatants were again filtered through Miracloth and centrifuged at 100,000×*g* for 90 min. The microsomal fraction (resulting pellets) were resuspended in 0.1 M potassium phosphate buffer with pH 7.2 and stored at − 80 °C until further usage for enzymatic assays. To determine the microsomal fraction concentration, aliquots of the suspensions were prepared and their phosphatidylcholine (PC) content was estimated.

Optimisation was carried out to establish the best in vitro assay condition of forward reaction of tested enzymes. The effect of various factors, such as the amount of microsomal fraction, reaction time, temperature, buffer pH and effect of selected ions (K^+^, Ca^2+^, Mg^2+^) on the activity of LPAAT and LPEAT type of enzymes (LPLATs involved in PA and PE biosynthesis with use of acyl-CoA and LPA or LPE, respectively) were investigated. The optimisation assays were performed with 5 nmol of [^14^C]18:1-CoA and 5 nmol of *sn*-1–18:1-lysophosphatidylethanolamine or *sn*-1–18:1-lysophosphatidic acid, respectively, for appropriate enzymes (LPEAT or LPAAT type of acyltransferases). In assays testing the effect of selected ions concentration HEPES buffer (pH 7.2) were used, because in potassium phosphate buffer, Mg^+2^ and Ca^+2^ ions can form insoluble salts. Microsomal fractions derived from *C. sativa* seeds at 24 DAF were used as a source of enzymes.

The detailed study of the substrate specificity of LPAAT and LPEAT type of enzymes of *C. sativa* seeds (in forward reactions) was performed with microsomal fractions derived from 17, 24 and 31 DAF with eight [^14^C]acyl-CoA: decanoyl-CoA ([^14^C]10:0-CoA), lauroyl-CoA ([^14^C]12:0-CoA), myristoyl-CoA ([^14^C]14:0-CoA), palmitoyl-CoA ([^14^C]16:0-CoA), stearoyl-CoA ([^14^C]18:0-CoA), oleoyl-CoA ([^14^C]18:1-CoA), linoleoyl-CoA ([^14^C]18:2-CoA) and linolenoyl-CoA ([^14^C]18:3-CoA). Assays were performed in a total volume of 100 µl 0.04 M potassium phosphate buffer (pH 7.2) containing 5 nmol of appropriate exogenous *sn*-1-18:1-lysophospholipids, 5 nmol of [^14^C]acyl-CoA and aliquots of microsomal fractions equivalent to 0.2 nmol or 0.5 nmol of microsomal PC (approximately 0.88 µg or 2.2 µg of microsomal proteins, respectively, for assays analysing LPAAT or LPEAT type of enzymes). Incubation of reaction mixtures was carried out at 30 °C for 30 and 60 min (respectively, for LPAATs and LPEATs assays) in Eppendorf Thermomixer Compact with continuous shaking (1250 rpm). In the substrate selectivity assays microsomal fraction derived from 31 DAF were used. 1 nmol of each of the five different acyl donors (one of them was [^14^C]labelled) was added to the reaction mixtures and all other conditions were as in assays performed with only one acyl donor as described above. There were two versions of the experiment: one without the addition of BSA, and the second one with the addition of 2 mg/ml of BSA.

The activity and substrate specificity of the tested enzymes of the developing seeds of *C. sativa* in the backward reactions were examined as described previously (Jasieniecka-Gazarkiewicz et al. 2016; Klińska et al. 2019). In these assays 10 nmol of [^14^C]acyl-CoA, 0.2 µmol of free coenzyme A (CoA) and 1 mg of BSA in a total volume of 100 µl of 0.04 M potassium buffer (pH 7.2) with or without 0.5 µmol of dithionitrobenzoic acid (DTNB) were used. Aliquots of microsomal fraction containing 5 nmol of endogenous PC were added into the reaction mixtures. Reactions were conducted for 60 min at 30 °C with shaking (1250 rpm). The activity of the backward reactions catalysed by the LPAAT and LPEAT type of enzymes of microsomal fractions of *C. sativa* seeds, was determined as described previously (Jasieniecka-Gazarkiewicz et al. 2016; Klińska et al. 2019).

Both forward and reverse reactions were terminated by addition of 375 µl chloroform:methanol (1:2, v:v), 5 µl of glacial acetic acid, 125 µl of chloroform and 125 µl of water. After shaking, the tubes were centrifuged (1000×*g*) for 2 min and the chloroform fractions (containing lipids) were collected and separated on TLC (Silica gel 60 plates; Merck) using chloroform:methanol:acetic acid:water (90:15:10:2,5; v:v:v:v) as the solvent system. Radiolabelled lipids ([^14^C]PA and [^14^C]PE) were visualised and quantified on the plates by electronic autoradiography (Instant Imager, Packard Instrument Co.).

### Lipid analyses

Extraction of lipids, from *C. sativa* seeds collected at 10, 17, 24, 31 and 60 DAF, as well as from prepared microsomal fractions, was conducted according to the modified method described by Bligh and Dyer (1959). Seeds/microsomes were homogenised in chloroform:methanol: 0.15 M acetic acid (1:2:1) in Potter–Elvehjem homogeniser and after addition of chloroform and water (in proportion 1:1; the volume equal to that from extraction) and subsequent mixing and centrifugation, the bottom chloroform fractions (containing lipids) were collected. To separate individual polar lipids classes, the obtained chloroform fractions were separated by TLC (Silica gel 60 plates; Merck) in chloroform:methanol:acetic acid:water (90:15:10:2,5; v:v:v:v) as the solvent system. Separated lipid classes were visualised by staining with a 0.05% primuline solution and visualisation under UV light. Areas of the gel containing the PC, PE and PA, identified by means of authentic standards, were scrapped off and lipids were transmethylated in situ on a gel by adding 2 ml of 2% H_2_SO_4_ in dry methanol (40 min at 90 °C). The obtained methyl esters were extracted with hexane; the internal standard of methyl-heptadecanoic acid was added and analyses were conducted by gas–liquid chromatography (Shimadzu; GC-2010) equipped with a flame ionization detector (FID) and a 60 m × 0.25 mm CP-WAX 58-CB fused-silica column (Agilent Technologies; Santa Clara, CA, USA).

## Results

### Phosphatidic acid (PA) and phosphatidylethanolamine (PE) in developing seeds of *C. sativa*

**Fig. 1:**
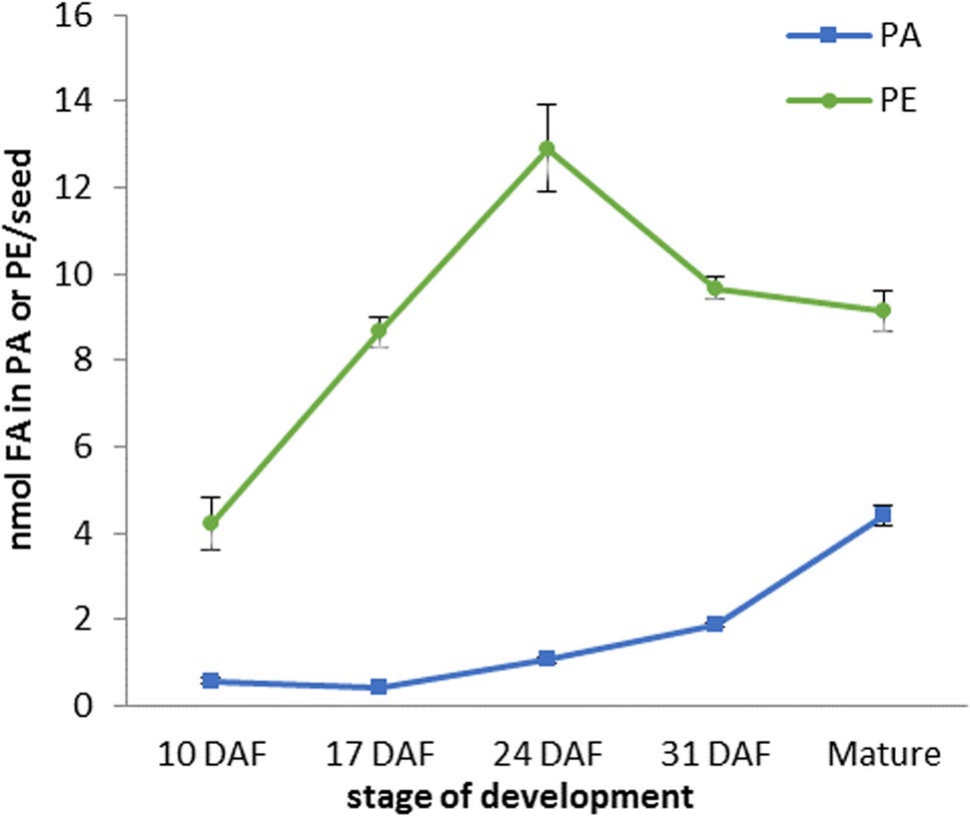
Content of phosphatidic acid (PA) and phosphatidylethanolamine (PE) in developing *Camelina sativa* seed. Mean values and SD are presented (*n* ≥ 3)

**Fig. 2:**
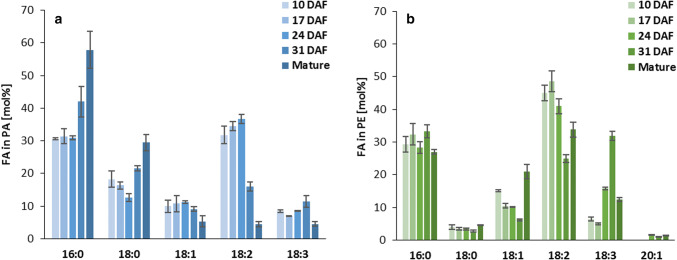
Fatty acids composition of phosphatidic acid (**a**) and phosphatidylethanolamine (**b**) in developing *Camelina sativa* seeds. Mean values and SD are given (*n* ≥ 3)

The content of phosphatidic acid (measured as the fatty acid content in this lipid per one seed) at early stages of seeds development were negligible; approximately 0.5 nmol FA (about 3% of total polar lipid content), but its amount increased gradually reaching 4.4 nmol FA in the mature seeds (about 9% of total polar lipids content) (Fig. 1 and Klińska et al. 2019). Palmitic (16:0) and linoleic (18:2) acids were the dominant fatty acid in PA until 24 DAF. Henceforth, the relative amount of 18:2 significantly decreased from 37 to 5% in mature seeds, while conversely the level of 16:0 increased from 31 to 58%. The relative amount of another saturated fatty acid—stearic acid—also increased after 24 DAF, reaching up to 29% at maturity (between 10 and 24 DAF its amount decreased from about 18 to 13%). Throughout the seeds development until 31 DAF the amount of oleic (18:1) and linolenic (18:3) acids ranged between 8–11% of all fatty acids of this lipid and dropped to about 4–5% in mature seeds (Fig. 2a).

The content of phosphatidylethanolamine in developing *C. sativa* seeds (measured as the fatty acid content in this lipid/ one seed) increased gradually between 10 and 24 DAF from about 4 to 13 nmol of FA. Afterwards the absolute amount of PE slightly declined to about 10 nmol FA. Up to 24 DAF, PE constitutes approximately 25% of all polar lipids and in later stages of seed development about 20% (Fig. 1 and Klińska et al. 2019). The relative amount of saturated fatty acids of PE of developing seeds of *C. sativa* was fairly stable during the entire seed development and fluctuated between 27–33% and 3–4%, for 16:0 and 18:0, respectively. In case of 18:1 and 18:2 tendencies for decrease were observed between 10 and 31 DAF (from 15 to 6% and from 45 to 25%, respectively), while the relative amount of both increased after that time and reached 21% and 34% in mature seeds. The opposite trend was detected for 18:3, which relative amount was growing from about 5–6% (10 and 17 DAF) up to 32% at 31 DAF, and after that time point decreased to 12% of all fatty acids of PE in mature seeds. Gondoic acid (20:1) was detected for the first time at 24 DAF and its maximum level accounted for 2% (Fig. 2b).

From the 31 DAF there was no net increase of total fatty acids in acyl-lipids of *C. sativa* seeds (Klińska et al. 2019). Thus, all of the changes in fatty acid composition observed between 31 DAF to complete maturity (60 DAF) were dependant only on endogenous conversion of these lipids including desaturases activity, acyltransferases activity, lipases activity and other reactions. The precise involvement of different biochemical pathways in this process cannot be, however evaluated.

### In vitro activity of *C. sativa* LPAAT and LPEAT type of enzymes (forward reactions)

**Fig. 3:**
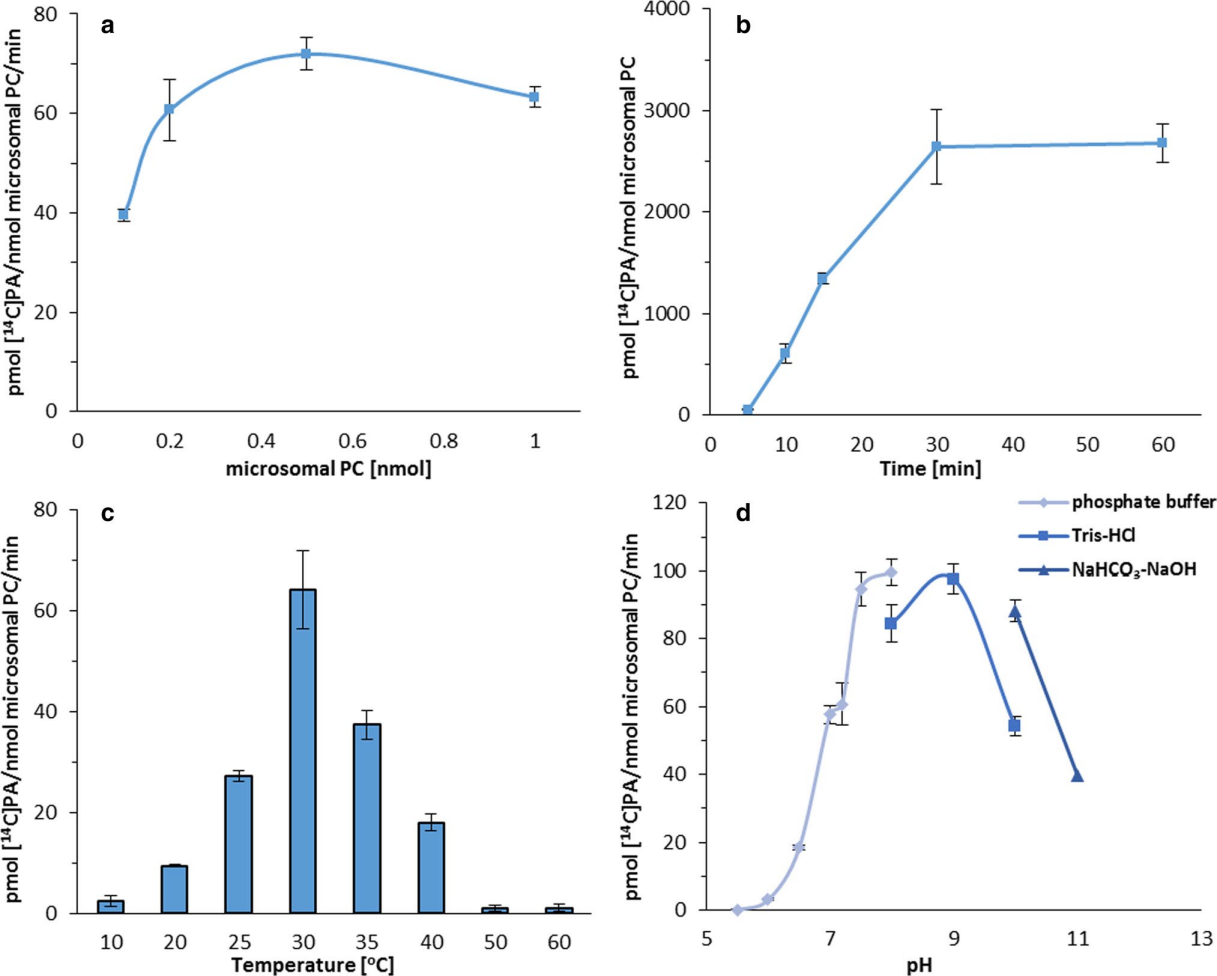
Effect of various factors on the activity of acyl-CoA:lysophosphatidic acid acyltransferases (LPAATs) of *Camelina sativa* seeds. **a** Microsomal content dependency. **b** Time dependency. **c** Temperature dependency. **d** pH dependency. Mean values and SD are presented (data from at least three independent assays)

**Fig. 4:**
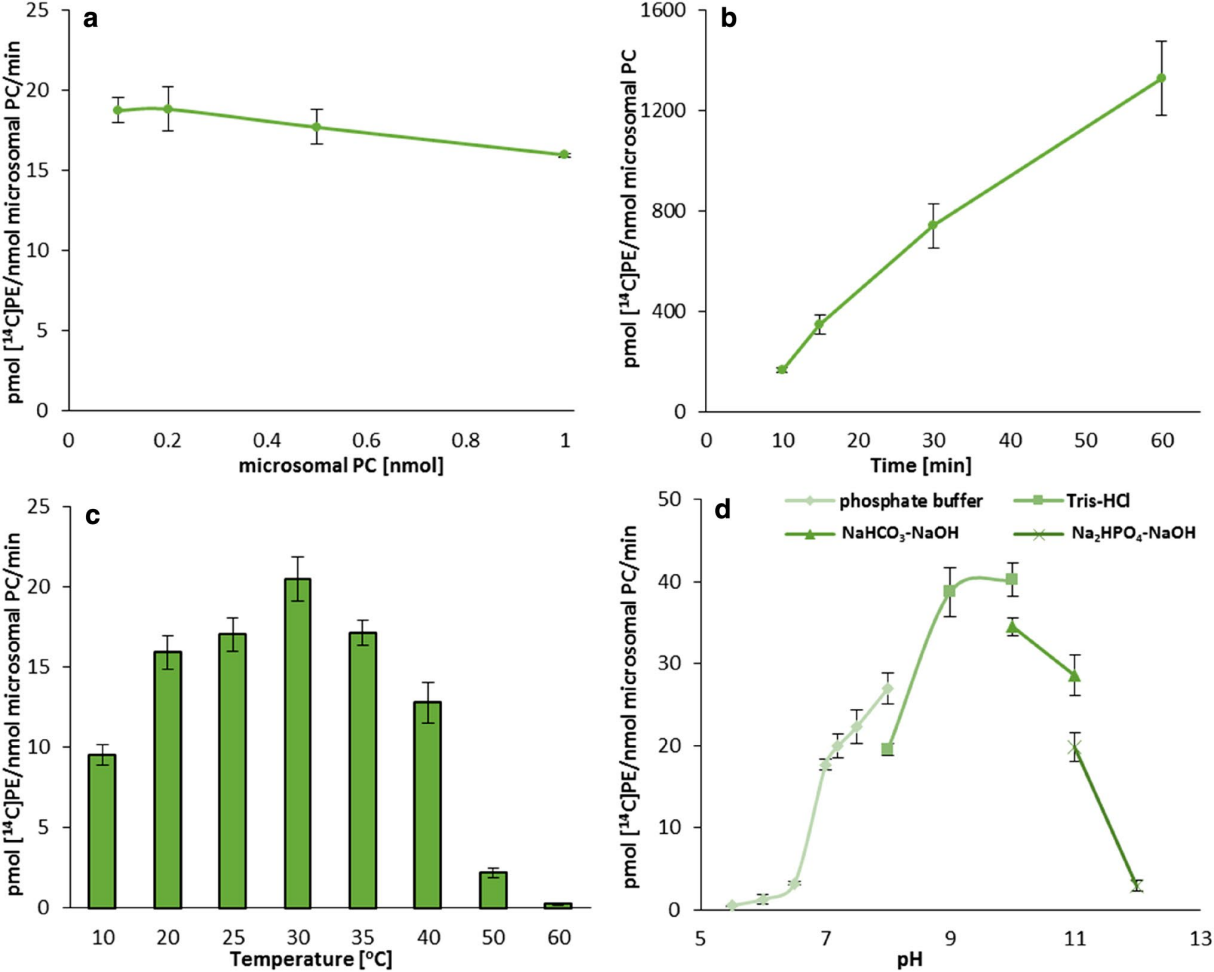
Effect of various factors on the activity of acyl-CoA:lysophosphatidylethanolamine acyltransferases (LPEATs) of *Camelina sativa* seeds. **a** Microsomal content dependency. **b** Time dependency. **c** Temperature dependency. **d** pH dependency. Mean values and SD are presented (data from at least three independent assays)

The optimal aliquots of microsomal fraction for assaying LPAAT type of enzymes activity were those containing 0.5 nmol of microsomal PC, and for assaying LPEAT type of enzymes 0.2 nmol of microsomal PC/assay. These values correspond to approximately 2.2 and 0.88 µg of microsomal proteins, respectively. Nevertheless, the activity of LPEAT type of enzymes did not differ markedly between the amounts of microsomal membrane from 0.1 to 1 nmol of microsomal PC. In case of LPAAT type of enzymes a decrease of microsomal membrane concentration in the assays to 0.1 nmol of microsomal PC/assay triggered the reduction of the activity up to 55% of its maximum level (Figs. 3a, 4a).

The time dependency assays indicated that the rate of reaction catalysed by the LPAAT type of enzymes was linear up to 30 min and plateaued afterwards. The activity of LPEAT type of enzymes maintained linearity up to 60 min (Figs. 3b, 4b). To get the best sensitivity of the performed assays (the highest amount of the product) we kept 30 and 60 min of the reaction time for LPAAT and LPEAT type of enzymes (respectively) in all subsequent assays. These reaction times were also implemented in the experiments evaluating the enzymes activity in different temperatures. In these experiments the amount of the reaction products could be affected both by the enzyme activity and denaturation rate. However, the denaturation rate of different enzymes could proceed with different intensity, and as far as each reaction runs linearly, it could be expected that the enzymes activity is the main factor determining the amount of reaction products. The linearity assays were performed at optimum temperature for the both tested enzymes.

Both tested type of enzymes reached their maximum activity at 30 °C. However, the activity of LPEAT type of enzymes changed only slightly when the temperature decreased to 20 °C or increased to 40 °C. On the contrary, in case of LPAAT type of enzymes even a modest decrease or increase of temperature by 5 °C, reduced the enzymes activity to about 42 and 58% of its maximum level, respectively. Decreasing the temperature to 10 °C resulted in decline of LPEAT type of enzymes activity to only about 1/2 of its maximum level, whereas LPAAT type of enzymes activity was marginal at that temperature. At the temperature above 50 °C, both analysed types of enzymes were almost inactive and in case of LPAAT type of enzymes it was true also for the temperature equal to 50 °C (Figs. 3c, 4c).

Three buffers with different pH ranges were used to determine the optimal values of pH for LPAAT type of enzymes activity: 0.1 M phosphate buffer (pH 5.5–8.0), 0.1 M Tris–HCl buffer (pH 8.0–10.0) and 0.1 M NaHCO_3_-NaOH buffer (pH 10.0 and 11.0). These three buffers were also used to determine the effect of pH on LPEAT type of enzymes activity, however, in these experiments an additional buffer (0.1 M Na_2_HPO_4_-NaOH buffer—pH 11.0 and 12.0) was also used as LPEAT type of enzymes maintain high activity in alkaline solutions. Both type of enzymes showed negligible activity at pH 5.0–5.5 (about 3% of their maximum activity). Increase of pH values caused significant raise of the tested enzymes activity. LPAAT type of enzymes recorded the maximum activity between pH 7.5–9.0 (phosphate and Tris–HCl buffers). The activity of this type of enzymes decreased to 55% of its maximum value at pH 10.0 when Tris–HCl buffer was used and only to 90% in case of NaHCO_3_-NaOH buffer. The strong inhibition of LPAAT type of enzymes activity in assays with the latter buffer was observed at pH 11.0 (the activity decreased to 40%). The optimal pH value for LPEAT type of enzymes ranges between 9.0–10.0 (Tris–HCl buffer). Further alkalisation of the reaction buffer leads to declining their activity; at pH 12.0 in Na_2_HPO_4_-NaOH buffer almost complete loss of the activity occurred (Figs. 3d, 4d). Although both enzymes reached their maximum activity at quite high pH, the further analyses were conducted at pH 7.2 as such pH value imitates condition closest to the natural environment of these enzymes.

The presence of Mg^2+^, Ca^2+^ and K^+^ ions in the incubation buffer affected the activity of the both tested type of enzymes. In these assays we used HEPES buffer due to formation of insoluble calcium and magnesium salts in phosphate buffer.

**Fig. 5:**
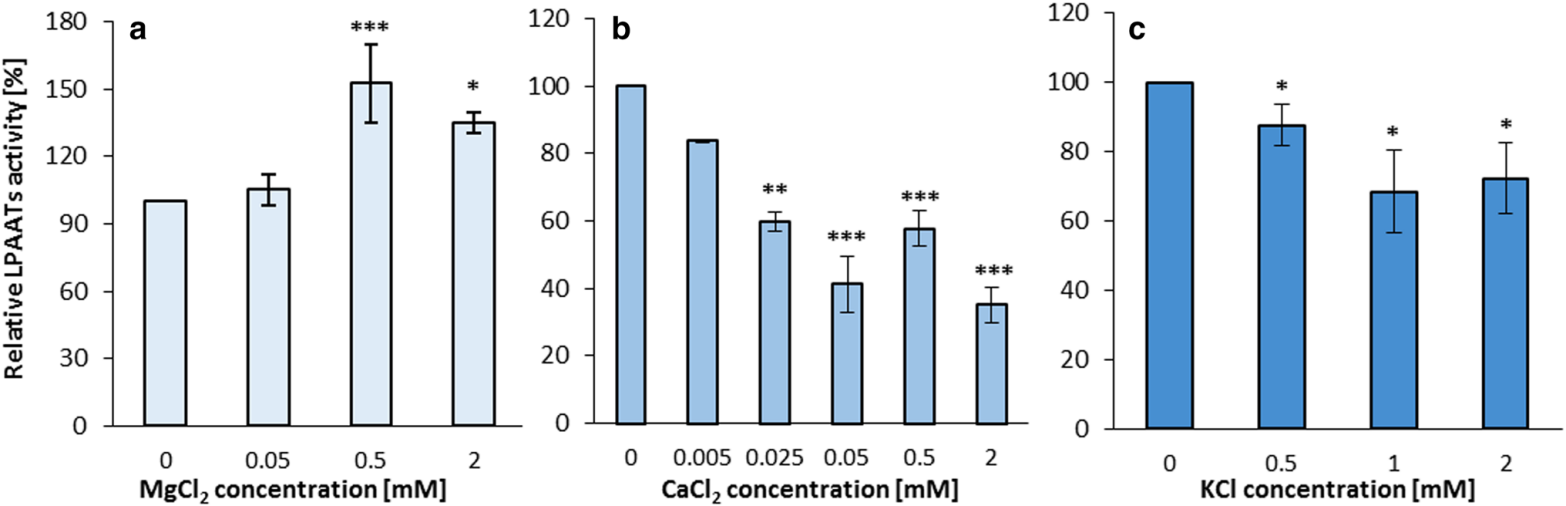
Effect of various ions on the activity of acyl-CoA:lysophosphatidic acid acyltransferases (LPAATs) of *Camelina sativa* seeds. **a** Effect of magnesium ions. **b** Effect of calcium ions. **c** Effect of potassium ions. Mean values and SD are presented (data from at least three independent assays). Asterisk denotes significant differences between control (ions not added) and tested ions concentrations in a mean difference two-sided Student’s *t* test: *p ≤ 0.05; **p ≤ 0.01; ***p ≤ 0.001

Addition of Mg^2+^ ions to the incubation buffer in 0.5–2.0 mM concentrations enhanced LPAAT type of enzymes activity by up to 40–50% compared to assays without these ions. Addition of Ca^2+^ and K^+^ ions produced opposite effects. These ions inhibited the activity of LPAAT type of enzymes and the inhibitory effect was stronger in case of calcium ions (compared with assays without Ca^2+^ the enzymes activity dropped down to 35%) (Fig. 5).

**Fig. 6:**
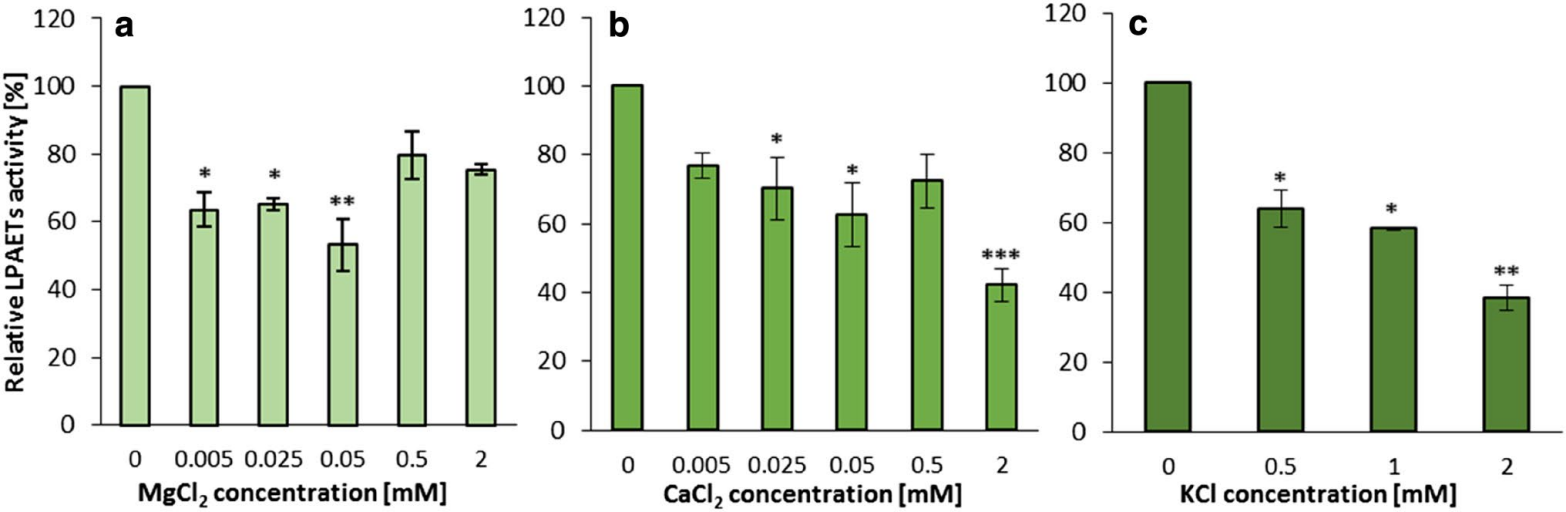
Effect of various ions on the activity of acyl-CoA:lysophosphatidylethanolamine acyltransferases (LPEATs) of *Camelina sativa* seeds. **a** Effect of magnesium ions. **b** Effect of calcium ions. **c** Effect of potassium ions. Mean values and SD are presented (data from at least three independent assays). Asterisk denotes significant differences between control (ions not added) and tested ions concentrations in a mean difference two-sided Student’s *t* test: *p ≤ 0.05; **p ≤ 0.01; ***p ≤ 0.001

In case of LPEAT type of enzymes the presence of all three tested ions in the incubation buffer inhibited their enzymatic activity. Addition of magnesium ions inhibited LPEAT type of enzymes activity by about 40% already in concentrations as low as 0.005 mM. The increase of Mg^2+^ ions concentration up to 10 times, changed their inhibitory effect only slightly. However, the increase of Mg^2+^ ions concentration to 0.5–2 mM resulted in reduction of its inhibitory effect (Fig. 6a). The calcium ions showed the inhibitory effect also at concentration 0.005 mM (about 25% inhibition) and the inhibition slightly increased with the increase of ions concentration. At 2 mM concentration Ca^2+^ ions inhibited the activity of LPEAT type of enzymes by about 60% (Fig. 6b). The presence of potassium ions in the incubation buffer at concentration of 0.5 mM inhibited the activity of LPEAT type of enzymes to 64% of its activity without the ions. Further increase of potassium ion concentration, strengthen the inhibitory effect. At concentration 2 mM only 39% of activity observed in assay without the ions were maintained (Fig. 6c).

### Activity and substrate specificity of LPAAT and LPEAT type of enzymes in developing *C. sativa* seeds in forward reactions

The activity of LPAAT and LPEAT type of enzymes was measured in microsomal fraction prepared from *C. sativa* seeds at 17, 24 and 31 DAF. Between these time points the lipids accumulation in *C. sativa* seeds is the most effective (Klińska et al. 2019). We used combinations of eight different exogenous [^14^C]acyl-CoAs with *sn-*1–18:1-LPA or *sn*-1–18:1-LPE to determine LPAAT and LPEAT type of enzymes substrate specificity, respectively.

The highest activity of LPAAT type of enzymes was observed in microsomal fraction derived from *C. sativa* seeds at 17 or 24 DAF, depending on acyl donors used in assays. At 17 DAF the highest activity was detected when 18:0-CoA and 18:1-CoA were applied, whereas at 24 DAF when 12:0-CoA, 16:0-CoA, 18:2-CoA and 18:3-CoA were tested. In assays with 10:0-CoA and 14:0-CoA the same activity was detected both at 17 and 24 DAF. The lowest activity of LPAAT type of enzymes was detected in microsomal fractions derived from seeds at 31 DAF for 7 out of 8 tested acyl-CoAs. Only in case of 18:2-CoA the lowest activity was detected in microsomal fractions from seeds at 17 DAF. Nevertheless, it should be noted, that the fluctuations in activity of LPAAT type of enzymes between analysed stages of *C. sativa* seeds development were not dramatic, and usually the lowest activity (for the majority of tested acyl-CoAs) reached at least 60% of the highest one.

**Fig. 7:**
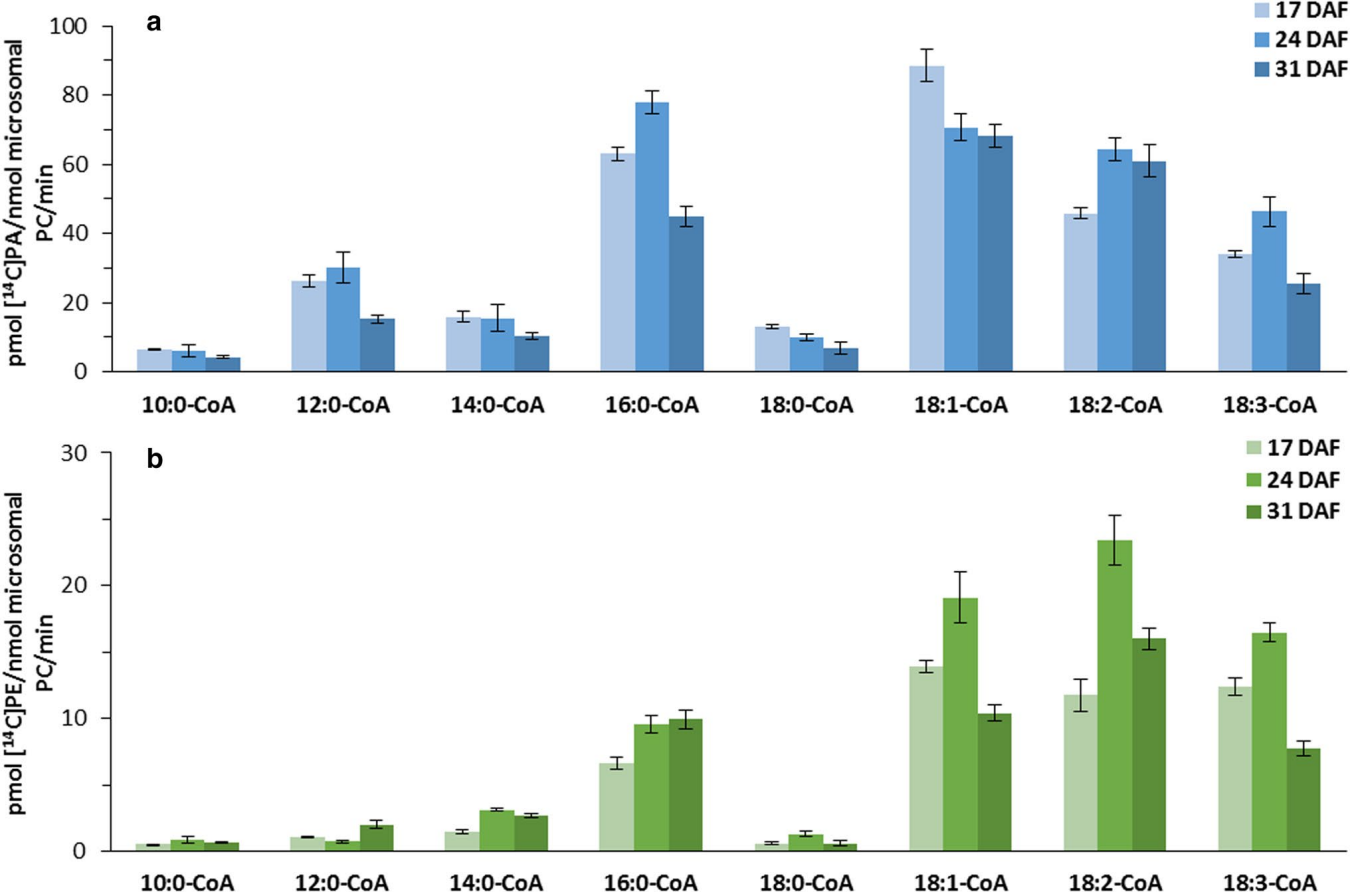
Activity of acyl-CoA:lysophosphatidic acid acyltransferases (LPAATs; **a**) and acyl-CoA:lysophosphatidylethanolamine acyltransferases (LPEATs; **b**) of *Camelina sativa* seeds towards 8 different acyl-CoAs (forward reaction). Mean values and SD are presented (data from at least three independent assays)

Unsaturated C18 acyl-CoAs and 16:0-CoA were the best accepted acyl donors by LPAAT type of enzymes. However, the preferences of these enzymes towards acyl donors changed somewhat with seed development. Among C18 unsaturated acyl-CoAs the 18:1-CoA was the most preferred one followed by 18:2-CoA and 18:3-CoA. Only at 24 DAF 16:0-CoA was better accepted by LPAAT type of enzymes than 18:1-CoA. The highest activity of LPAAT type of enzymes, obtained in assays with microsomal fractions prepared at 17 DAF and 18:1-CoA as acyl donor amounted to 78 pmol [^14^C]PA/nmol of microsomal PC/min (about 20 nmol [^14^C]PA/mg microsomal protein/min). Besides the acyl-CoAs mentioned earlier, LPAAT type of enzymes accepted also 10:0-CoA, 12:0-CoA, 14:0-CoA and 18:0-CoA, however, with lower intensity. Among these acyl donors 12:0-CoA was the best one with the activity ranging between 22 to 42%, of the activity towards 18:1-CoA (Fig. 7a).

The highest activity of LPEAT type of enzymes was detected in microsomal fraction prepared from seeds at 24 DAF for six out of eight tested acyl-CoAs. The two exceptions concern 12:0-CoA and 16:0-CoA which when applied as acyl donors give the highest activity at 31 DAF. At least half of the maximum activity was maintained in assays with microsomes of two remaining stages of *C. sativa* seeds development for all the tested acyl donors.

Unsaturated 18C-acyl-CoAs were the most preferred acyl donors for the reaction catalysed by LPEAT type of enzymes. 16:0-CoA was also well accepted. At first analysed stage, 18:1-CoA was the favoured acyl donor, but during the next two stages it was dethroned by 18:2-CoA. 18:3-CoA was utilised with the efficiency ranging between 50 to 70% and 16:0-CoA with 43 to 63% of activity towards 18:2-CoA, respectively. The activity of the remaining combinations of the tested substrates was negligible, below 13% of activity towards 18:2-CoA (Fig. 7b).

The preferences of both LPAAT and LPEAT type of enzymes of *C. sativa* seeds microsomal fractions for different acyl-CoAs observed in single acyl-CoA assays (described above) were verified in substrate specificity assays. In these assays, reaction mixtures were supplemented with 5 different acyl-CoAs in equimolar amount (1 nmol of each, wherein one of them was [^14^C]-labelled).

**Fig. 8:**
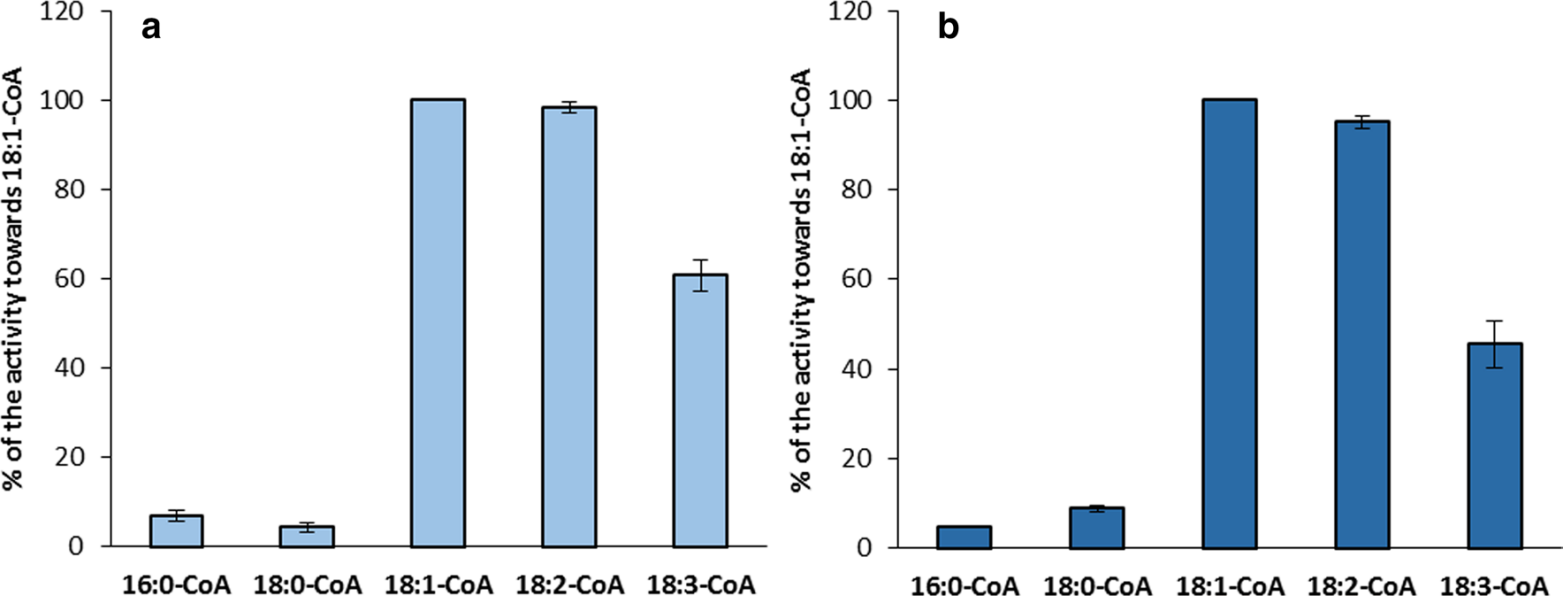
Activity of acyl-CoA:lysophosphatidic acid acyltransferases (LPAATs) of *Camelina sativa* seeds towards five different acyl-CoAs added to the reaction mixture together in equimolar concentrations without (**a**) or with (**b**) the addition of BSA (forward reaction; substrate selectivity assay). For the assays microsomal fractions of seeds from 31 DAF were used. Mean values and SD are presented (data from at least three independent assays)

In these assays LPAAT type of enzymes showed almost equal preference towards 18:1-CoA and 18:2-CoA, and about 40% lower efficiency towards 18:3-CoA. Thus, the activity towards unsaturated 18C acyl-CoA was very similar to the preferences of these enzymes obtained in the single acyl-CoA assays. In case of saturated acyl-CoAs the preferences of LPAAT type of enzymes was, however, much lower. Both 16:0-CoA and 18:0-CoA were utilised with only about 5 and 4% (respectively) efficiency expressed by these enzymes towards 18:1-CoA. On the contrary, in the single acyl-CoA assays 16:0-CoA was used with about 70% efficiency and 18:0-CoA with about 7% efficiency of the utilisation of 18:1-CoA. Addition of BSA to the incubation buffer did not influence significantly the utilisation of the tested acyl-CoAs (Fig. 8a, b).

**Fig. 9:**
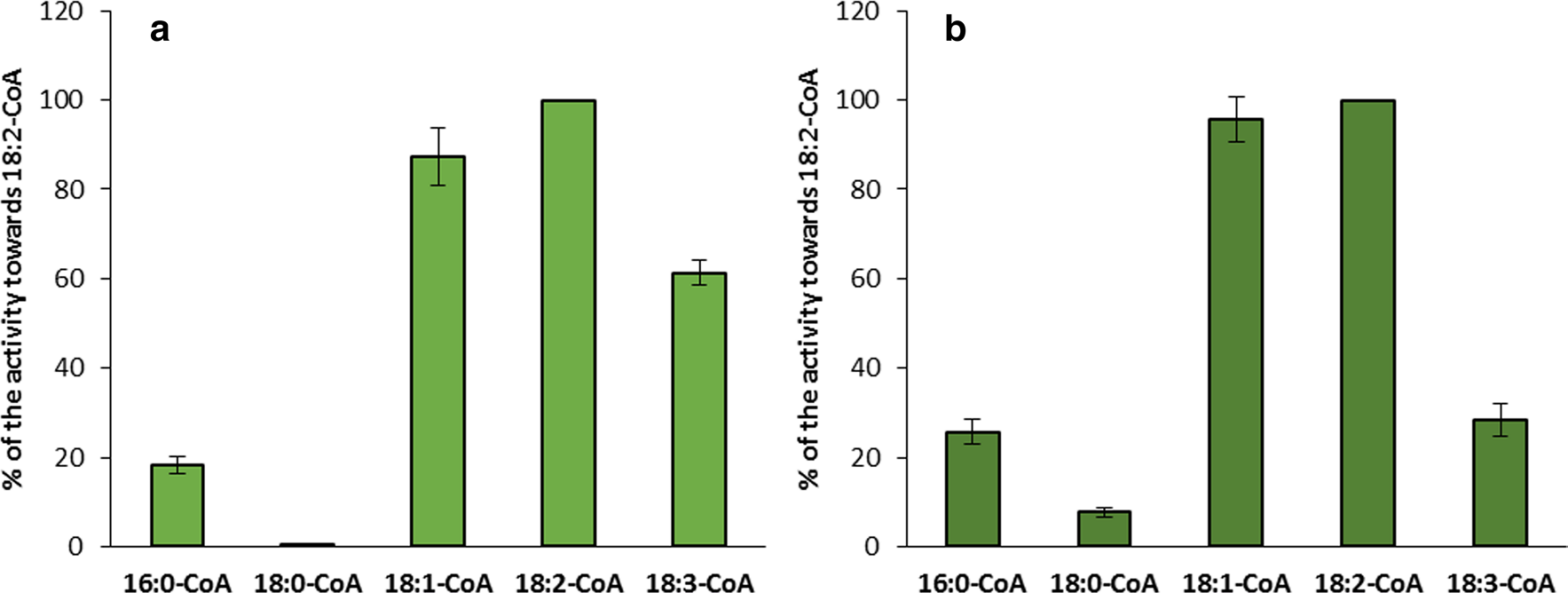
Activity of acyl-CoA:lysophosphatidylethanolamine acyltransferases (LPEATs) of *Camelina sativa* seeds towards five different acyl-CoAs added to the reaction mixture together in equimolar concentrations without (**a**) or with (**b**) the addition of BSA (forward reaction; substrate selectivity assay). For the assays microsomal fractions of seeds from 31 DAF were used. Mean values and SD are presented (data from at least three independent assays)

Similarly to the situation described above also the preferences of LPEAT type of enzymes for unsaturated 18C acyl-CoA were resembling the single acyl-CoA assays and the preferences towards 16:0-CoA and 18:0-CoA was much lower. Addition of BSA to the incubation buffer does not materially alter these preferences. However, some decreases in utilisation of 18:3-CoA and a slightly better utilisation of saturated acyl-CoAs were noted (Fig. 9a, b).

### Activity and substrate specificity of LPAAT and LPEAT type of enzymes in developing *C. sativa* seeds in backward reactions

Considering that not only LPCATs (acyl-CoA:lysophosphatidylcholine acyltransferases) but also LPAAT and LPEAT type of acyltransferases show the ability to conduct the backward reaction (Jasieniecka-Gazarkiewicz et al. 2016), we have measured it in microsomal fractions of *C. sativa* seeds. We used [^14^C]-labelled 18:1-CoA, 18:2-CoA and 18:3-CoA as fatty acids donors for remodelling PA or PE of microsomal fractions of *C. sativa* seeds. Exogenous lysophospholipids were not added to the reaction mixture as they would trigger the production of [^14^C]PA or [^14^C]PE in the forward reaction. They were only produced from endogenous phospholipids via the backward reaction of acyl-CoA:lysophospholipid acyltransferases—LPLATs) or via the other type of reaction catalysed, e.g., by phospholipases or PDAT during the reaction time. Addition of DTNB to the reaction mixture binds free coenzyme A and in this way stops the backward reactions catalysed by LPLATs. The assays were carried out with and without addition of DTNB. By subtracting the amount of de novo synthetized [^14^C]phospholipid in the presence of DTNB, from the amount of [^14^C]phospholipid synthetized in the reaction without DTNB we get the amount of de novo synthesised [^14^C]phospholipid via the LPLAT action (for detail see: Jasieniecka-Gazarkiewicz et al. 2016 and Klińska et al. 2019).

The intensity of de novo synthesis of [^14^C]PA and [^14^C]PE in assays with *C. sativa* seeds microsomal fractions via the action of LPLAT (acyl-CoA:lysophospholipid acyltransferases), calculated in the way described above, was treated as the backward reaction activity of LPAAT and LPEAT (respectively) type of enzymes.

The backward reaction intensity catalysed by LPAAT type of enzymes was the highest in assays with microsomal fractions prepared from *C. sativa* seeds at 24 DAF and the lowest in assays with microsomes prepared at 31 DAF. The used acyl donors affected also that activity; the highest activity was observed when 18:1-CoA was in the incubation mixtures, followed by 18:2-CoA (except for 31 DAF) and 18:3-CoA. In case of 18:1-CoA as a fatty acid donor for remodelling PA the backward reaction activity at 17 DAF maintained at least 62% and in 31 DAF -31% of the maximum activity observed at 24 DAF. When 18:2-CoA and 18:3-CoA were used, at 24 DAF the activity with these acyl-CoAs was about 19% and 69% (respectively) lower than with 18:1-CoA. The activity with 18:2-CoA dropped to about a half both in 17 DAF and 31 DAF and the activity with 18:3-CoA was similar at 17 DAF and fell to 1/3 at 31 DAF.

The intensity of remodelling of PE via the backward reaction of LPEAT type of enzymes, at each of the analysed stages, was the highest with the presence of 18:2-CoA in the assays, although along with development time, activity was declining, maintaining at 31 DAF only 1/4 of its activity at 17 DAF. Gradual reduction of activity was also observed in case of the two other acyl donors: 18:1-CoA and 18:3-CoA, wherein at 31 DAF, their participation in remodelling of PE by LPEAT type of enzymes was no longer observed (Table 1).

**Table 1:** Incorporation of [^14^C]acyl groups from [^14^C]acyl-CoA into PA and PE of microsomal fractions of *Camelina sativa* seeds in the presence or absence of DTNB

Stage of development	acyl-CoA used in the assay	LPAAT pmol [^14^C]PA/nmol microsomal PC/min			LPEAT pmol [^14^C]PE/nmol microsomal PC/min		
		− DTNB	+ DTNB	∆	− DTNB	+ DTNB	∆
17 DAF	[^14^C]18:1-CoA	0.18 ± 0.03	0	0.18	0.23 ± 0.02	0.12 ± 0.01	0.11
	[^14^C]18:2-CoA	0.12 ± 0.01	0	0.12	0.36 ± 0.03	0.2 ± 0.003	0.16
	[^14^C]18:3-CoA	0.08 ± 0.003	0	0.08	0.16 ± 0.017	0.12 ± 0.01	0.04
24 DAF	[^14^C]18:1-CoA	0.31 ± 0.02	0.02 ± 0.001	0.29	0.12 ± 0.018	0.06 ± 0.006	0.06
	[^14^C]18:2-CoA	0.26 ± 0.02	0.03 ± 0.004	0.23	0.23 ± 0.008	0.1 ± 0.008	0.13
	[^14^C]18:3-CoA	0.12 ± 0.01	0.03 ± 0.004	0.09	0.09 ± 0.009	0.06 ± 0.004	0.03
31 DAF	[^14^C]18:1-CoA	0.17 ± 0.018	0.08 ± 0.005	0.09	0.08 ± 0.007	0.10 ± 0.001	0
	[^14^C]18:2-CoA	0.18 ± 0.018	0.07 ± 0.003	0.11	0.12 ± 0.01	0.08 ± 0.019	0.04
	[^14^C]18:3-CoA	0.08 ± 0.013	0.05 ± 0.003	0.03	0.04 ± 0.001	0.05 ± 0.0	0

The delta depicts incorporation with DTNB subtracted from incorporation without DTNB and represents the incorporation via acyl exchange (LPAAT and LPEAT type of enzymes backward reaction)

In case of remodelling of PA in assays with microsomal fraction of *C. sativa* seeds, the participation of “other reactions–Lands cycle” (assays with addition of DTNB) was negligible or very low at 17 and 24 DAF (respectively). However, at 31 DAF the participation of “other reactions” and backward reaction of LPAAT type of enzymes was more or less equal. In remodelling of PE both types of reactions participated almost equally at 17 and 24 DAF, whereas at 31 DAF the participation of backward reaction of LPEAT type of enzymes were negligible (Table 1).

### Potential transfer of fatty acids from PA and PE to acyl-CoA pool or directly to TAG

**Fig. 10:**
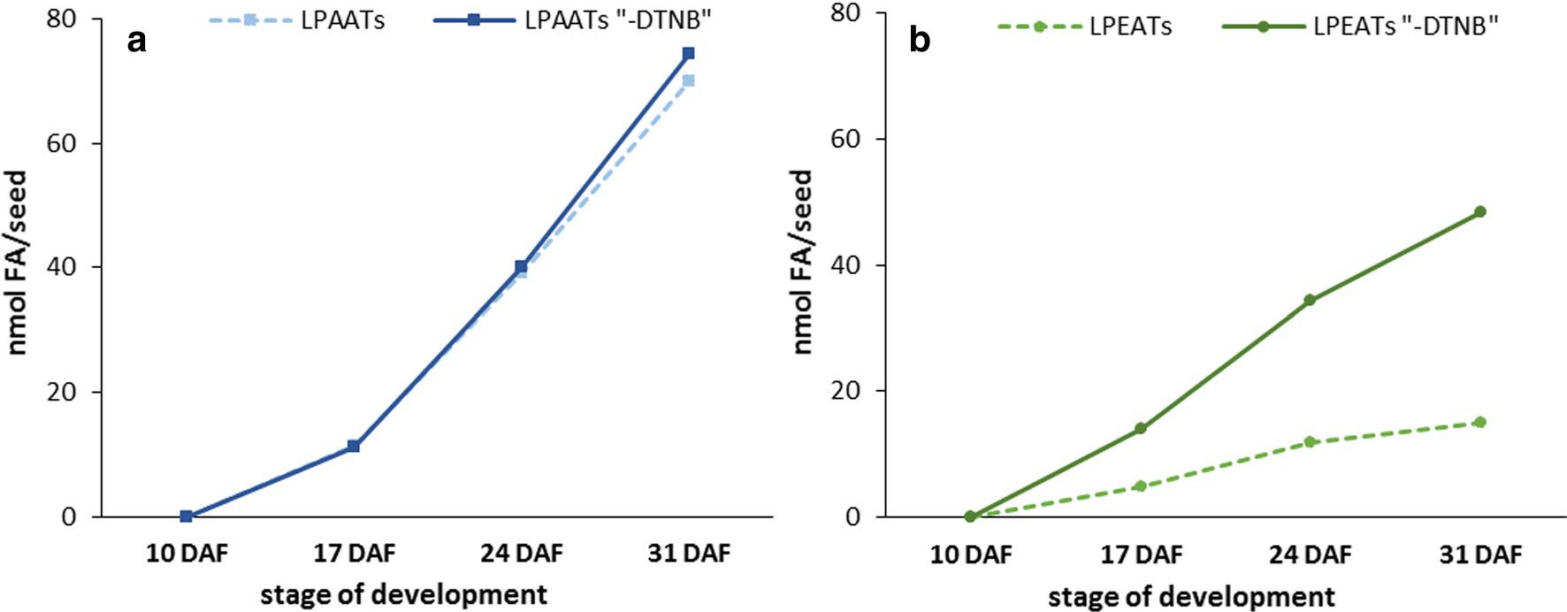
Potential amount of fatty acids transferred from phosphatidic acid or phosphatidylethanolamine to acyl-CoA pool via backward reaction catalysed by *Cs*LPAATs (**a**) or *Cs*LPEATs (**b**) and by these enzymes and other enzymes (calculated based on backward reaction activity in vitro). Mean values and SD are given (*n* ≥ 3)

Polyunsaturated fatty acids (18:2 and 18:3) account for up to 55% of all fatty acids in PA and up to 60% in PE (Fig. 2). During the remodelling of these phospholipids via the action of LPLAT type of enzymes 18:2 and 18:3 can be transferred to acyl-CoA pool, where they are available either for synthesis of, e.g., TAG or remodelling of other lipids. The potential amount of fatty acids (including polyunsaturated fatty acids) that could be transferred from PA or PE to acyl-CoA pool was calculated based on the backward reaction intensity of LPAAT and LPEAT type of enzymes in a way described previously (Klińska et al. 2019). To estimate the total activity of the tested enzymes per 1 seed we used the activity of backward reactions obtained from assays with [^14^C]18:1-CoA as a fatty acid donor for remodelling of PA or PE (pmol [^14^C]PA or pmol [^14^C]PE/nmol microsomal PC/min) and multiplied it by the amount of nmol PC present in 1 seed (according to Klińska et al. 2019). Then, we took the time span between analysed stages of seed development (in minutes) and multiplied it by an average value of the total activities from two adjacent points in time (for the time period between 0 to 17 DAF we used half of the activity at 17 DAF) of the tested enzymes in 1 seed. As a result we got the amount of fatty acids that could be transferred from the PA or PE to acyl-CoA pool via backward reactions of the mentioned enzymes between the analysed stages of seed development. In the same way we calculated the potential transfer of fatty acids from these phospholipids via LPLATs backward reaction and “other reaction”/Lands cycle. However, instead of LPLAT backward reaction activity (value: Δ from Table 1) we used the intensity of PA or PE remodelling obtained in assays without DTNB (value: -DTNB from Table 1), (Fig. 10).

The potential transfer of fatty acids from PA to the acyl-CoA pool is presented on Fig. 10a. This transfer occurred mainly via action of LPAAT type of enzymes. In this way about 10 nmol FA could be transferred from PA of one seed until 17 DAF, about 40 nmol FA until 24 DAF and over 70 nmol of fatty acids until 31 DAF. This constitutes about 5% of fatty acids of all acyl lipids present in the seeds of *C. sativa* at each of these stages of development (Klińska et al. 2019). “Other reaction”/Lands cycle including phospholipases (FA removed from PA can be esterified to CoA) or PDAT (direct transfer to TAG) accounted for only a couple of percentage points of the total transfer, and occurred only between 24 and 31 DAF.

The transfer of fatty acids from PE to acyl-CoA pool via LPEAT type of enzymes action is low and amounts to approximately 5, 10 and 13 nmol after 17, 24 and 31 DAF, respectively (Fig. 10b). This constitutes only 2.2, 1.1 and 1 percentage of fatty acids of all lipids of C. *sativa* seeds at respective stages of development (Klińska et al. 2019). Contrary to PA the potential transfer of fatty acids from PE via “other reaction”/Lands cycle was higher and accounted for approximately 10, 23 and 31 nmol after 17, 24 and 31 DAF, respectively (Fig. 10b). The transfer of fatty acids from PE via both types of reaction accounted for 6.8, 3.7 and 3.4 percentage of fatty acids of all lipids presented in *C. sativa* seeds at 17, 24 and 31 DAF, respectively (Klińska et al. 2019).

### The time of complete fatty acids turnover in PC, PE and PA of *C. sativa* seeds

The intensity of exogenous [^14^C]fatty acids incorporation into PA, PE (results presented in this paper) and PC (Klińska et al. 2019) in assays with microsomal fractions from *C. sativa* seeds at different stages of development computed earlier for the measurement of the backward reaction intensity of LPLAT allowed also for calculation of the potential time of complete fatty acids turnover in theses lipids. The amounts of fatty acids in PA, PE or PC per one seeds (Fig. 1 and Klińska et al. 2019) presented in pmol were divided by an estimated total activity in remodelling PA, PE and PC (via LPLAT backward reaction and “other reaction”) in one seed (calculated as described above, using data from assays without DTNB; value: -DTNB from Table 1 for PA and PE and for PC from our previous publication: Klińska et al. 2019; Table 4). In this way we got an expected time necessary for exchange of all acyl groups (in *sn-1* and *sn-2* positions) in a given phospholipid for fatty acids from acyl-CoA pool. As fatty acids from *sn-2* position are preferentially utilised by the reactions leading to the remodelling of the tested phospholipids (Lager et al. 2013), the estimated time for complete exchange of fatty acids from position *sn-2* is probably shorter and from position *sn-1* longer than calculated average time of complete remodelling of fatty acids in these lipids.

The most efficient remodelling of fatty acids occurred in case of PA. The complete turnover of fatty acids in this lipid ranged between 1.9 to 6.9 h when 18:1-CoA or 18:2-CoA were used as fatty acid donors. In case of 18:3-CoA the time was longer and ranged from 4.8 to 14.7 h. The slowest efficiency in fatty acids remodelling was observed in PE. The time for complete turnover ranged from 24.6 to 169.2 h. Also in case of this lipid the efficiency of fatty acids remodelling was the slowest when 18:3-CoA was used as fatty acid donor. The time for complete remodelling of fatty acids in PC ranged between 5.3 and 12 h and the remodelling was the most efficient (like in case of PE) when 18:2-CoA was used as the fatty acid donor (in case of PA the shortest time was observed when 18:1-CoA was the fatty acids donor). (Table 2).

**Table 2:** Estimated time of complete exchange of acyl groups of PE, PA and PC for fatty acids from acyl-CoA pool (calculated based on the intensity of incorporation of acyl group from [^14^C]acyl-CoA into PA, PE and PC in vitro) at different stages of *Camelina sativa* seeds development

Fatty acid donors	Stage of development	Exchange time of acyl group of PA, PE and PC for acyl group from acyl-CoA pool [h]		
		PA	PE	PC
[^14C^]18:1-CoA	17 DAF	2.4	38.7	5.3
	24 DAF	1.9	60.2	8.2
	31 DAF	6.9	77.8	9.4
[^14^C]18:2-CoA	17 DAF	3.5	24.6	6.3
	24 DAF	2.2	30.9	7.7
	31 DAF	6.4	51.2	6.5
[^14^C]18:3-CoA	17 DAF	5.2	55.6	9.4
	24 DAF	4.8	78.9	12.0
	31 DAF	14.7	169.2	11.0

## Discussion

Thus far, fatty acids remodelling was mainly studied on phosphatidylcholine (PC) and there are only scarce data concerning other plant phospholipids (Lager et al. 2013; Renne et al. 2016; Jasieniecka-Gazarkiewicz et al. 2016). There are two pathways of remodelling of phospholipids: the reactions connected with the Lands’ cycle and the reactions connected with the LPLAT cycle (**see Introduction). The Lands’ cycle postulates involvement of two types of enzymes: one producing lysophospholipids (LPLs), e.g., by phospholipases and the other synthesising new molecules of phospholipids by acyl-CoA:lysophospholipid acyltransferases (LPLATs). On the other hand, in the LPLAT cycle both creation of lysophospholipids and reacylation of these molecules are performed via LPLATs action in the backward and forward reactions, respectively. In this study we evaluated the involvement of both cycles in the remodelling of phosphatidic acid (PA) and phosphatidylethanolamine (PE) of microsomal fractions of developing *C. sativa* seeds. The LPLATs involved in the synthesis of [^14^C]PA with use of LPA and [^14^C]acyl-CoA we called LPAAT type of enzymes and that one synthesised [^14^C]PE from LPE and [^14^C]acyl-CoA LPEAT type of enzymes. In the synthesis of [^14^C]PA and [^14^C]PE the LPA and LPE present in the assays can be utilised not only by *Cs*LPAATs and *Cs*LPEATs (respectively) but also by other LPLATs to synthesise PA or PE (Jasieniecka-Gazarkiewicz et al. 2016). Consequently, we cannot pinpoint to what extent *Cs*LPAATs or *Cs*LPEATs are responsible for the synthesis of [^14^C]PA or [^14^C]PE in our assays as the microsomal fractions contain different types of LPLATs (*Cs*LPCATs, *Cs*LPAATs and *Cs*LPEATs). However, judging by the substrate specificity and biochemical proprieties of *Cs*LPCATs presented by Klińska et al. (2019), as well as substrate specificity and biochemical proprieties of LPAATs type of enzymes and LPEATs type of enzymes obtained in this work, we can argue that in the biosynthesis of [^14^C]PC, [^14^C]PA and [^14^C]PE in the assays with microsomal fractions of *C. sativa* developing seeds different classes of LPLATs play the dominant role. Therefore, it is quite likely that in the de novo synthesis of [^14^C]PC *Cs*LPCAT plays the dominant role (such a conclusion was also drown by Klińska et al. 2019), while in the synthesis of [^14^C]PA, the dominant role can be attributed to *Cs*LPAATs and in the synthesis of [^14^C]PE to *Cs*LPEATs. Thus the substrate specificity and the biochemical properties of *Cs*LPLATs called so far the “LPAAT type of enzymes” are probably very close or identical to the properties of *Cs*LPAATs and those of “LPEAT type of enzymes” to the properties of *Cs*LPEATs (with just marginal modifications caused by “other *Cs*LPLATs” in both cases). Thus, in the remaining part of the discussion we will just refer to *Cs*LPAATs and *Cs*LPEATs.

To obtain a more detailed characterisation of *Cs*LPAATs and *Cs*LPEATs additional studies are needed. The genes should be cloned and transferred to other organism lacking these type of genes, e.g., yeast with knocked-out main LPLAT – ALE1 (for details see Jasieniecka-Gazarkiewicz et al. 2016). Another strategy could involve preparation of *C. sativa* mutants lacking *Cs*LPCATs and *Cs*LPEATs to study *Cs*LPAATs; and mutants lacking *Cs*LPCATs and *Cs*LPAATs to study *Cs*LPEATs. However, it is impossible to predict if such mutants will be viable. Purification of *Cs*LPEATs or/and *Cs*LPAATs proteins probably will not be an effective method as both enzymes are membrane bound and purification of such proteins usually results in obtaining not active enzymes.

LPAATs are acyltransferases from the Kennedy pathway with the highest substrate stringency (Laurent and Huang 1992). It has been shown that their substrate specificity towards acyl-CoA can vary substantially in different plant species (Oo and Huang 1989; Lassner et al. 1995; Arroyo-Caro et al. 2013; Fahs et al. 2019). Presented research shows that *Cs*LPAATs have the highest preferences towards unsaturated [18C]acyl-CoA (especially towards 18:1-CoA) and 16:0-CoA. However, in substrate selectivity assays 16:0-CoA was discriminated (in contrasts to similar assays, especially with addition of BSA, with *Cs*LPCATs Klińska et al. 2019), while unsaturated [18C]acyl-CoA was utilised with similar preferences as in single acyl-CoA assays. This occurred both in case of assays without and with BSA (which in in vitro conditions mimics the effects of ACBPs in vivo—both can bind acyl-CoAs and reduce availability of the free acyl-CoAs for the acyltransferases—Stymne and Stobart 1984; Engeseth et al. 1996). Consequently, in in vivo conditions unsaturated [18C]acyl-CoA are probably mostly used as fatty acid donors for PA synthesis catalysed by *Cs*LPAATs. As 18:1-CoA is the dominant component of acyl-CoA pool of *C. sativa* seeds (Ruiz-Lopez et al. 2016) we should expect that 18:1 could be a major component of fatty acids of this lipid. Nevertheless, fatty acids of PA of *C. sativa* seeds contained only about 10% of 18:1. This means that additional mechanism is involved in regulation of the quality of PA species remaining in the membranes of *C. sativa* seeds.

So far only the substrate specificity of LPEAT of *A. thaliana* has been studied (Stålberg et al. 2009 and Jasieniecka-Gazarkiewicz et al. 2016). These papers showed that *At*LPEAT has very high preferences towards 16:0-CoA and unsaturated [18C]acyl-CoA. Substrate specificity of *Cs*LPEATs reflects to some degree the above mentioned finding, with one caveat that we recorded a slightly lower affinity towards 16:0-CoA. The preferences of *Cs*LPEATs towards 16:0-CoA diminished further in substrate selectivity assays, however, to a lower degree than in case of *Cs*LPAATs discussed above. Thus this phenomenon is probably not only connected with differences in solubility of various acylo-CoA but also with the proprieties of tested enzymes. The fatty acid composition of PE of *C. sativa* seeds does not fully reflect the preferences of *Cs*LPEATs towards the tested acyl-CoAs. This lipid is, however, very slowly remodelled, thus the composition of fatty acids of DAG used for its synthesis could have probably the highest effect on its fatty acid composition.

In addition to the higher relative affinity (in single assays test) of *Cs*LPAATs and *Cs*LPEATs towards 16:0-CoA in comparison with *Cs*LPCATs, the first two classes of LPLATs show also a relatively higher efficiency (especially *Cs*LPAATs) towards 10:0-CoA, 12:0-CoA and 14:0-CoA than *Cs*LPCATs (presented data and Klińska et al. 2019).

All three types of *Cs*LPLATs showed the highest activity at 30 °C, however, they reacted differently to temperature changes. *Cs*LPEATs were the most resistant to the temperature changes; their activity did not differ considerably between 20 and 40 °C. The biggest changes of activity (connected with the temperature) were recorded in case of *Cs*LPAATs. Even a small increase or decrease of the temperature by 5 °C considerably reduced their activity. Reaction of *Cs*LPCATs to the temperature changes was somewhere in between the two extreme cases described above (presented data and Klińska et al. 2019). The tested temperatures included both those occurring in a real-life environment (10 °C do 40 °C), and more extreme ones (50 and 60 °C). Form all the discussed LPLATs, *Cs*LPAATs could probably have the strongest effects on net lipid synthesis as the Kennedy pathway enzymes. Thus, one can speculate, that a strong dependency of the activity of these enzymes on the temperature could affect lipid biosynthesis in vivo, depending on the temperature in which plants are growing. This hypothesis needs, however, experimental backing.

Changes in pH affected the activity of all three types of *Cs*LPLATs in slightly similar manner. All of them were almost inactive up to pH 6.0 and their activity was rather high in alkaline pH; at least up to pH 11.0. However, in case of *Cs*LPCATs the highest activity was recorded for the pH between 7.0 and 11.0 and the changes in activity in this range of pH were insignificant. The highest activity of *Cs*LPAATs was obtained for pH 7.5 to pH 9.0 and of *Cs*LPEATs between pH 9.0 and pH 10.0 (presented data and Klińska et al. 2019). One can conclude that each of these classes of acyltransferases have their own unique pattern of shifts in activity in response to changes in pH. The range of tested pH was probably much wider than existing in plant cells. The most probable pH inside the cells’ cytosol is around pH 7. Thus, in the assays examining forward and backward substrate specificity of the tested enzymes we used pH 7.2. Nevertheless, in certain compartments of cells the pH could differ from cytosolic one. The tested broad range of pH was significant, however, for the biochemical characterisation of the tested enzymes.

The effect of the addition of magnesium, calcium and potassium ions on *Cs*LPCATs, *Cs*LPAATs and *Cs*LPEATs activity depended on the analysed class of acyltransferases. *Cs*LPCATs activity was inhibited by all three tested ions and the decrease in activity was the highest in case of Ca^2+^ ions and the lowest in case of K^+^ ions (Klińska et al. 2019). *Cs*LPEATs activity was also inhibited by all three tested ions, however, the highest inhibitory effect was recorded for K^+^ ions. Any increase of Mg^2+^ ions concentration above 0.05 mM instead of further decreasing the enzymes activity (like in case of *Cs*LPCATs), gave a lower inhibitory effect. In case of *Cs*LPAATs, Mg^2+^ ions maintained or stimulated the activity, while Ca^2+^ ions and K^+^ ions have inhibitory effect (especially Ca^2+^). Stimulatory effect of Mg^2+^ ions on LPAAT of *Brassica napus, Cocos nucifera, Limnanthes alba, Syagrus cocoides* and *Zea mays* activity have already been reported by others (Oo and Huang 1989; Cao et al. 1990; Davies et al. 1995). Both in our work and in the work mentioned above, we observed that the increase of Mg^2+^ ions concentration over a certain point does not further increase the enzymes activity, and could even have an inhibitory effect. This could probably be explained by the fact that at higher concentrations Mg^2+^ ions adversely affect acyl-CoA solubility (Constantinioles and Steim 1986). The effect of Ca^2+^ ions on LPAAT activity of microsomal fractions of palm endosperm, maize scutellum and rapeseed cotyledon have been examined earlier by Oo and Huang (1989) and similarly to our findings they reported Ca^2+^ inhibitory effect. Contrary to our work with *Cs*LPEAT, LPEAT of *Arabidopsis thaliana*, which was cloned into the yeast system produced no or minor response to Ca^2+^ (Stålberg et al. 2009). We did not find any research on the effect of K^+^ ions on LPLAT activity in other plants.

Total Mg^2+^ concentration in cells range from 15 to 25 mM (Moomaw and Maguire 2008). However, most Mg^2+^ ions are bound or incorporated into cellular components, which leaves only about 0.4 to 0.5 mM free cytosolic Mg^2+^ (Karley and White 2009; Maathuis 2009). The Ca^2+^ ions concentration in the cytoplasm could be as low as 1 µM, however in some compartments it can reach mM levels (Hepler 2005; Bagur and Hajnóczky 2017). K^+^ ions concentration in plant cells remains relatively high; up to 200 mM in the cytosol (Blumwald el al. 2000). The range of ions concentration tested in these studies (Mg^2+^—0.05 to 2 mM; Ca^2+^—0.005 to 2 mM; K^+^—0.5 to 2 mM) was in most cases within natural levels. Thus, the results obtained in vitro can most probably reflect those prevailing in nature.

The data in the genomic databases of *C. sativa* indicate the presence of five LPAAT isoenzymes (LPAAT1, LPAAT2, LPAAT3, LPAAT4 and LPAAT5) with each of them probably occurring in three isoforms. In case of LPEATs two isoenzymes are hypothesised, with potential for three isoforms of each of them (Camelina Genome Project Portal; https://camelinagenomics.org). Until now, however, no experimental data have confirmed that these genes can truly encode *Cs*LPAATs or *Cs*LPEATs. Based on these predictions, preliminary study on expression of LPAATs in developing seeds of *C. sativa* has been made by Abdullah et al. (2016). Their results show that between the first stage of seed development (10–15 DAF) and the second one (16–21 DAF), the expression levels of LPAAT1-4 candidate genes increase, while the expression level of LPAAT5 decrease. Analysis of the expression levels of LPEAT isoenzymes does not exist for any plant tissue, *Camelina sativa* including. However, similarly to LPAATs and LPCATs hypothesised isoforms (Abdullah et al. 2016), also in LPEATs isoforms we should expected variations in expression levels during *C. sativa* seed development. The observed differences in affinity of *Cs*LPAATs and *Cs*LPEATs towards the tested acyl-CoAs at particular stages of seeds development could probably be explained by the different expression of individual isoforms of these enzymes in the tested stages.

Phosphatidic acid (PA) constitutes only a small fraction of all polar lipid of *C. sativa* seeds (about 2–4% up to 31 DAF and about 9% at mature seeds). In spite of this, its contribution in supplying the cytosolic acyl-CoA pool with acyl-CoA seems to be relatively high. Our data indicate that about 5% of fatty acids present in mature *C. sativa* seeds are first esterified with PA, then transferred to acyl-CoA pool via* Cs*LPAATs action and later reused for lipid biosynthesis or remodelling. This is a surprising result as PA is not the place for fatty acid desaturation. PA also constitutes a far too small fraction of membrane lipids for fatty acid composition of its molecules to significantly affect the membrane fluidity. Phosphatidic acid is, nevertheless, a substrate for phosphatidic acid phosphatases providing DAGs for different kinds of syntheses. These DAG molecules are for instance utilised for PC synthesis in which fatty acid desaturation takes place (Li-Beisson et al. 2013). It is, therefore, possible that fast remodelling of fatty acids composition of PA and preferential utilisation of 18:1-CoA in this process create a mechanism for supplying DAG molecules enriched with 18:1 for PC synthesis. Whether PA species enriched with 18:1 are preferentially utilised by phosphatidic acid phosphatases remains a question for further investigation. However, a low amount of 18:1 in PA extracted/present in *C. sativa* seeds supports such a possibility.

Contrary to PA, remodelling of phosphatidylethanolamine (PE) is very slowly and its contribution to supplying the cytosolic acyl-CoA pool with acyl-CoA via the action of *Cs*LPEATs seems to be relatively low. Our results indicate that it could amount to only about 2% of the final fatty acids content in acyl-lipids of mature *C. sativa* seeds in spite of the fact that PE contributes to approximately 25% of all polar lipids. Nevertheless, remodelling of PE via the “other reaction / Lands cycle” could be higher than via* Cs*LPEAT action. In our assays both types of reactions had almost identical contribution, however in vivo “other reaction” could produce much higher values; PE is the best fatty acid donor for TAG biosynthesis via PDAT action (Ståhl et al. 2004). As a by-product of this reaction LPE is formed and could be reacylated with acyl-CoA from cytosolic acyl-CoA pool by LPEATs. However, our assays did not measure PDAT activity in the tested microsomal fractions and eventual evaluation of PDAT contribution to PE remodelling remains for further study.

### *Author contributions statement*

All authors conceived and designed the research. SK, KJ-G and KD conducted the experiments. SK, KJ-G, KD and AB analysed the data and wrote the manuscript. All authors read and approved the manuscript.

## Abbreviations

DAF: Days after flowering
DAG: Diacylglycerol
DTNB: Dithionitrobenzoic acid
FA: Fatty acid
LPA: Lysophosphatidic acid
LPE: Lysophosphatidylethanolamine
LPCAT: Acyl-CoA:lysophosphatidylcholine acyltransferase
LPAAT: Acyl-CoA:lysophosphatidic acid acyltransferases
LPEAT: Acyl-CoA:lysophosphatidylethanolamine acyltransferases
LPLAT: Acyl-CoA:lysophospholipid acyltransferases
PA: Phosphatidic acid
PE: Phosphatidylethanolamine
PC: Phosphatidylcholine
TAG: Triacylglycerol
10:0: Capric acid
12:0: Lauric acid
14:0: Myristic acid
16:0: Palmitic acid
18:0: Stearic acid
18:1: Oleic acid
18:2: Linoleic acid
18:3: α-Linolenic acid
20:1: Gondoic acid

## References

[CR1] Abdullah HM, Akbari P, Paulose B, Schnell D, Qi W, Park Y, Pareek A, Dhankher OP (2016). Transcriptome profiling of *Camelina sativa* to identify genes involved in triacylglycerol biosynthesis and accumulation in the developing seeds. Biotechnol Biofuels.

[CR2] Arroyo-Caro JM, Chileh T, Kazachkov M, Zou J, Alonso DL, García-Maroto F (2013). The multigene family of lysophosphatidate acyltransferase (LPAT)-related enzymes in *Ricinus communis*: cloning and molecular characterization of two LPAT genes that are expressed in castor seeds. Plant Sci.

[CR3] Bafor M, Smith MA, Jonsson L, Stobart K, Stymne S (1991). Ricinoleic acid biosynthesis and triacylglycerol assembly in microsomal preparations from developing castor-bean (*Ricinus communis*) endosperm. Biochem J.

[CR4] Bafor M, Smith MA, Jonsson L, Stobart K, Stymne S (1993). Biosynthesis of vernoleate (cis-12-epoxyoctadeca-cis-9-enoate) in microsomal preparations from developing endosperm of *Euphorbia lagascae*. Arch Biochem Biophys.

[CR5] Bagur R, Hajnóczky G (2017). Intracellular Ca^2+^ sensing: role in calcium homeostasis and signalling. Mol Cell.

[CR6] Banaś A, Bafor M, Wiberg E, Lenman M, Ståhl U, Stymne S, Williams JP, Khan MU, Lem NW (1997). Biosynthesis of an acetylenic fatty acid in microsomal preparations from developing seeds of *Crepis alpina*. Physiology, biochemistry and molecular biology of plant lipids.

[CR7] Bates PD, Ohlrogge JB, Pollard M (2007). Incorporation of newly synthesized fatty acids into cytosolic glycerolipids in pea leaves occurs *via* acyl editing. J Biol Chem.

[CR8] Bates PD, Stymne S, Ohlrogge J (2013). Biochemical pathways in seed oil synthesis. Curr Opin Plant Biol.

[CR9] Bligh EG, Dyer WJ (1959). A rapid method of total lipid extraction and purification. Can J Biochem Physiol.

[CR10] Blumwald E, Aharon G, Apse M (2000). Sodium transport in plant cells. Biochem Biophys Acta.

[CR11] Cao YZ, Oo KC, Huang AHC (1990). Lysophosphatidate acyltransferase in the microsomes from maturing seeds of meadowfoam (*Limnanthes alba*). Plant Physiol.

[CR12] Constantinides PP, Steim JM (1986). Solubility of palmitoyl-coenzyme A in acyltransferase assay buffers containing magnesium ions. Arch Biochem Biophys.

[CR13] Davies HM, Hawkins DJ, Nelsen JS (1995). Lysophosphatidic acid acyltransferase from immature coconut endosperm having medium chain length substrate specificity. Phytochemistry.

[CR14] Engeseth NJ, Pacovskya RS, Newman T, Ohlrogge JB (1996). Characterization of an acyl-CoA-binding protein from *Arabidopsis thaliana*. Arch Biochem Biophys.

[CR15] Fahs Z, Rossez Y, Guénin S, Gutierrez L, Thomasset B, Perrin Y (2019). Cloning and molecular characterization of three lysophosphatidic acid acyltransferases expressed in flax seeds. Plant Sci.

[CR16] Griffiths G, Stymne S, Stobart AK (1988). The utilisation of fatty-acid substrates in triacylglycerol biosynthesis by tissue-slices of developing safflower (*Carthamus tinctorius* L.) and sunflower (*Helianthus annuus* L.) cotyledons. Planta.

[CR17] Hepler PK (2005). Calcium: a central regulator of plant growth and development. Plant Cell.

[CR18] Jasieniecka-Gazarkiewicz K, Demski K, Lager I, Stymne S, Banaś A (2016). Possible role of different yeast and plant lysophospholipid:acyl-CoA acyltransferases (LPLATs) in acyl remodelling of phospholipids. Lipids.

[CR19] Karley AJ, White PJ (2009). Moving cationic minerals to edible tissues: potassium, magnesium, calcium. Curr Opin Plant Biol.

[CR20] Kennedy EP (1961). Biosynthesis of complex lipids. Fed Proc.

[CR21] Kennedy EP, Weiss SB (1956). The function of cytidine coenzymes in the biosynthesis of phospholipides. J Biol Chem.

[CR22] Klińska S, Jasieniecka-Gazarkiewicz K, Banaś A (2019). Acyl-CoA:lysophosphatidylcholine acyltransferases (LPCATs) of *Camelina sativa* seeds – biochemical properties and function. Planta.

[CR23] Lager I, Yilmaz JL, Zhou XR, Jasieniecka K, Kazachkov M, Wang P, Zou J, Weselake R, Smith MA, Bayon S, Dyer JM, Shockey JM, Heinz E, Green A, Banaś A, Stymne S (2013). Plant acyl-CoA:lysophosphatidylcholine acyltransferases (LPCATs) have different specificities in their forward and reverse reactions. J Biol Chem.

[CR24] Lands WE (1958). Metabolism of glycerolipides; a comparison of lecithin and triglyceride synthesis. J Biol Chem.

[CR25] Lassner MW, Levering CK, Davies HM, Knutzon DS (1995). Lysophosphatidic acid acyltransferase from meadowfoam mediates insertion of erucic acid at the *sn*-2 position of triacylglycerol in transgenic rapeseed oil. Plant Physiol.

[CR26] Laurent P, Huang AHC (1992). Organ- and development-specific acyl coenzyme A lysophosphatidate acyltransferases in palm and meadowfoam. Plant Physiol.

[CR27] Li-Beisson Y, Shorrosh B, Beisson F, Andersson MX, Arondel V, Bates PD, Baud S, Debono A, Durrett TP (2013). Acyl-lipid metabolism. Arabidopsis Book.

[CR28] Maathuis FJM (2009). Physiological functions of mineral macronutrients. Curr Opin Plant Biol.

[CR29] Moomaw AS, Maguire ME (2008). The unique nature of Mg^2+^ channels. Physiology.

[CR30] Ohlrogge J, Browse J (1995). Lipid biosynthesis. Plant Cell.

[CR31] Ohlrogge JB, Kuhn DN, Stumpf PK (1979). Subcellular-localization of acyl carrier protein in leaf protoplasts of *Spinacia oleracea*. PNAS.

[CR32] Oo KC, Huang AH (1989). Lysophosphatidate acyltransferase activities in the microsomes from palm endosperm, maize scutellum, and rapeseed cotyledon of maturing seeds. Plant Physiol.

[CR33] Pan X, Chen G, Kazachkov M, Greer MS, Caldo KM, Zou J, Weselake RJ (2015). In vivo and in vitro evidence for biochemical coupling of reactions catalyzed by lysophosphatidylcholine acyltransferase and diacylglycerol acyltransferase. J Biol Chem.

[CR34] Pollard M, Ohlrogge J (1999). Testing models of fatty acid transfer and lipid synthesis in spinach leaf using in vivo oxygen-18 labelling. Plant Physiol.

[CR35] Renne MF, Bao X, De Smet CH, de Kroon AIPM (2016). Lipid acyl chain remodeling in yeast. Lipid Insights.

[CR36] Ruiz-Lopez BR, Usher S, Salas JJ, Haslam RP, Napier JA, Beaudoin F (2016). Tailoring the composition of novel wax esters in the seeds of transgenic *Camelina sativa* through systematic metabolic engineering. Plant Biotechnol J.

[CR37] Sánchez M, David GN, David NB (1973). The relationship between palmitoyl-coenzyme A synthetase activity and esterification of *sn*-glycerol 3-phosphate in rat liver mitochondria. Biochem J.

[CR38] Ståhl U, Carlsson AS, Lenman M, Dahlqvist A, Huang B, Banaś W, Banaś A, Stymne S (2004). Cloning and functional characterization of a phospholipid: diacylglycerol acyltransferase from *Arabidopsis*. Plant Physiol.

[CR39] Stålberg K, Ståhl U, Stymne S, Ohlrogge J (2009). Characterization of two *Arabidopsis thaliana* acyltransferases with preference for lysophosphatidylethanolamine. BMC Plant Biol.

[CR40] Stymne S, Glad G (1981). Acyl exchange between oleoyl-CoA and phosphatidylcholine in microsomes of developing soya bean cotyledons and its role in fatty acid desaturation. Lipids.

[CR41] Stymne S, Stobart AK (1984). The biosynthesis of triacylglycerols in microsomal preparations of developing cotyledons of sunflower (*Helianthus annuus* L.). Biochem J.

[CR42] Williams JP, Imperial V, Khan MU, Hodson JN (2000). The role of phosphatidylcholine in fatty acid exchange and desaturation in *Brassica napus* L. leaves. Biochem J.

